# Lessons learned from Integrated Management Program Advancing Community Treatment of Atrial Fibrillation (IMPACT-AF): a pragmatic clinical trial of computerized decision support in primary care

**DOI:** 10.1186/s13063-021-05488-y

**Published:** 2021-08-11

**Authors:** Joanna M. Nemis-White, Laura M. Hamilton, Sarah Shaw, James H. MacKillop, Ratika Parkash, Shurjeel H. Choudhri, Antonio Ciaccia, Feng Xie, Lehana Thabane, Jafna L. Cox, for the IMPACT-AF Investigators

**Affiliations:** 1 Principal, Strive Health Management Consulting Ltd., Halifax, Nova Scotia Canada; 2 Research Manager, QEII Health Sciences Centre, Nova Scotia Health Authority, Halifax, Nova Scotia Canada; 3 Healthy Communities Program Officer, Public Health, Nova Scotia Health Authority, Halifax, Nova Scotia Canada; 4 Family Physician, Sydney Primary Care Medical Clinic, Sydney, Nova Scotia Canada; 5 Division of Cardiology, Department of Medicine, Dalhousie University, Halifax, Nova Scotia Canada; 6 Senior Vice President and Head, Medical & Scientific Affairs, Bayer Inc, Mississauga, Ontario Canada; 7 Director & Head, Medical Affairs – Cardiovascular Medicine, Bayer Inc, Mississauga, Ontario Canada; 8 Professor, Department of Health Research Methods, Evidence, and Impact, McMaster University, Hamilton, Ontario Canada; 9 Centre for Health Economics and Policy Analysis, McMaster University, Hamilton, Ontario Canada; 10 Vice President, Research, St. Joseph’s Healthcare, Hamilton, Ontario Canada; 11 Professor, Departments of Anesthesia/Pediatrics, McMaster University, Hamilton, Ontario Canada; 12 Director, Biostatistics Unit, Centre for Evaluation of Medicine, McMaster University, Hamilton, Ontario Canada; 13 Senior Scientist, Population Health Research Institute (PHRI), Hamilton Health Sciences, McMaster University, Hamilton, Ontario Canada; 14 Department of Community Health and Epidemiology, Dalhousie University, Halifax, Nova Scotia Canada; 15 Heart and Stroke Foundation of Nova Scotia Endowed Chair in Cardiovascular Outcomes Research, Halifax, Nova Scotia Canada

**Keywords:** Atrial fibrillation, Clinical trials, Informatics, Clinical decision support

## Abstract

**Background:**

Integrated Management Program Advancing Community Treatment of Atrial Fibrillation (IMPACT-AF) was a pragmatic, cluster randomized trial assessing the effectiveness of a clinical decision support (CDS) tool in primary care, Nova Scotia, Canada. We evaluated if CDS software versus Usual Care could help primary care providers (PCPs) deliver individualized guideline-based AF patient care.

**Methods:**

Key study challenges including CDS development and implementation, recruitment, and data integration documented over the trial duration are presented as lessons learned.

**Results:**

*Adequate resources must be allocated for software development, updates and feasibility testing*. Development took longer than projected. End-user feedback suggested network access and broadband speeds impeded uptake; they felt further that the CDS was not sufficiently user-friendly or efficient in supporting AF care (i.e., repetitive alerts).

*Integration across e-platforms is crucial*. Intellectual property and other issues prohibited CDS integration within electronic medical records and provincial e-health platforms. Double login and data entry were impediments to participation or reasons for provider withdrawal. Data integration challenges prevented easy and timely data access, analysis, and reporting.

*Primary care study recruitment is resource intensive*. Altogether, 203 PCPs and 1145 of their patients participated, representing 25% of eligible providers and 12% of AF patients in Nova Scotia, respectively. The most effective provider recruitment strategy was in-office, small group lunch-and-learns. PCPs with past research experience or who led patient consent were top recruiters. The study office played a pivotal role in achieving patient recruitment targets.

**Conclusions:**

A rapid growth in healthcare data is leading to widespread development of CDS. Our experience found practical issues to address for such applications to succeed. Feasibility testing to assess the utility of any healthcare CDS prior to implementation is recommended. Adequate resources are necessary to support successful recruitment for future pragmatic trials. CDS tools that integrate multiple co-morbid guidelines across eHealth platforms should be pursued.

**Trial registration:**

ClinicalTrials.gov NCT01927367. Registered on August 22, 2013

## Background

Atrial fibrillation (AF) is a common chronic condition associated with increased mortality, substantial morbidity, and high health care costs [1]. It is also an independent risk factor for stroke and, as such, clinical guidelines recommend antithrombotic therapy for stroke prevention in most patients with AF [2]. Despite national guidelines, gaps in provider knowledge and patient care have been documented [3, 4]_._

Integrated Management Program Advancing Community Treatment of Atrial Fibrillation (IMPACT-AF) was a pragmatic, cluster randomized trial assessing the clinical relevance and effectiveness of a clinical decision support (CDS) tool in the primary care setting of Nova Scotia, Canada [5]. The IMPACT-AF study methods and main results have been published [5, 6]. Briefly, clinical and health informatics researchers developed and evaluated if CDS software could help primary care providers (PCPs) better deliver individualized AF patient care based on national guidelines, thereby improving process of care and patient health outcomes at 12 months. No significant effects on the primary efficacy or safety outcomes were observed at follow-up [6].

### Need for documentation of lessons learned

The use of electronic medical records (EMRs) has been increasing among community-based clinicians. CDS tools have begun to appear as well, although few of those available in Canada and elsewhere have been validated through randomized controlled trials [7–10]. As such, limited information has been published on the potential challenges that might arise when developing and implementing a point-of-care decision support software application in a clinical trial setting, whether for atrial fibrillation or any other medical condition. The purpose of this paper is to offer insight into the key challenges faced in the development, implementation, and assessment of a CDS software, with lessons learned that would support the success of future work in this area of practice. We also provide suggestions for other researchers based on our experience and learnings.

## Methods

**Fig. 1:**
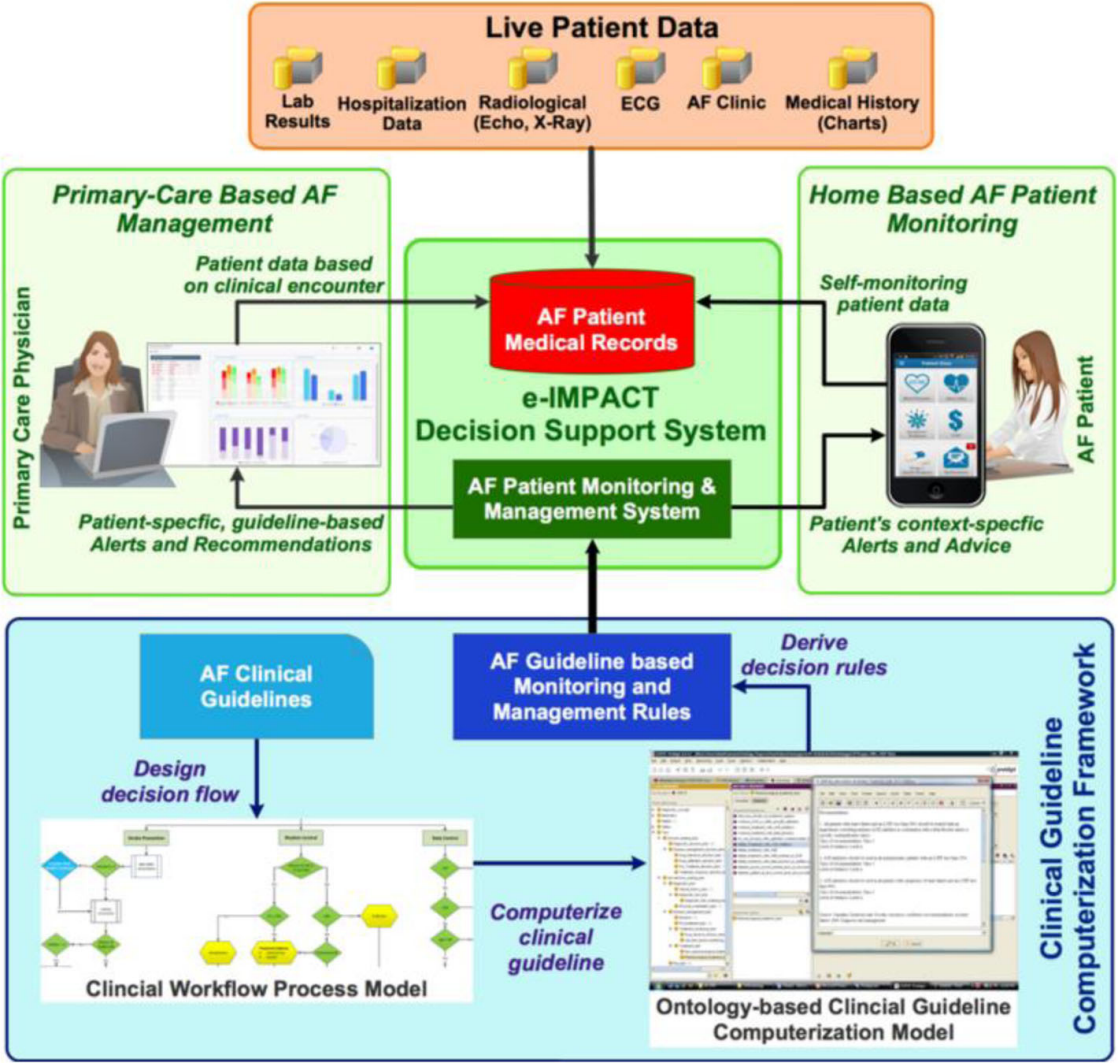
CDS design. AF, atrial fibrillation; ECG, electrocardiogram

Following ethics approval by the Nova Scotia Health Authority Research Ethics Board, software design began in 2013. The goal was to create a fully integrated application that computerized national clinical guidelines into decision rules to support AF patient management at point-of-care (Fig. 1). A key CDS feature was prioritized automated alerts signaling material changes in patient clinical or biochemical profiles requiring expedited treatment modifications. Users, both providers and patients, could interact with the software by entering and receiving health-related information and or alerts. Where possible, relevant patient data could be captured in real time (Fig. 1) [11].

From 2014 to 2016, PCPs and their patients with AF were identified, recruited, and randomized 1:1 to CDS access (*n* = 104 PCPs, *n* = 597 patients) or usual care (*n* = 99 PCPs, *n* = 548 patients). Consented patients were followed for 12 months with assessments of process of care and health outcomes conducted. Throughout the study duration, the research team documented key challenges regarding CDS development and implementation (2013–2018), data integration (2013 to 2020), and study recruitment (2014–2016).

## Results

### Lessons learned

#### CDS-related challenges

*Key lessons: Sufficient resources must be allocated for software development, feasibility testing, and software updates, and the integration across e-platforms is crucial for CDS use and study data (timely access, analysis, and reporting).*

**Fig. 2:**
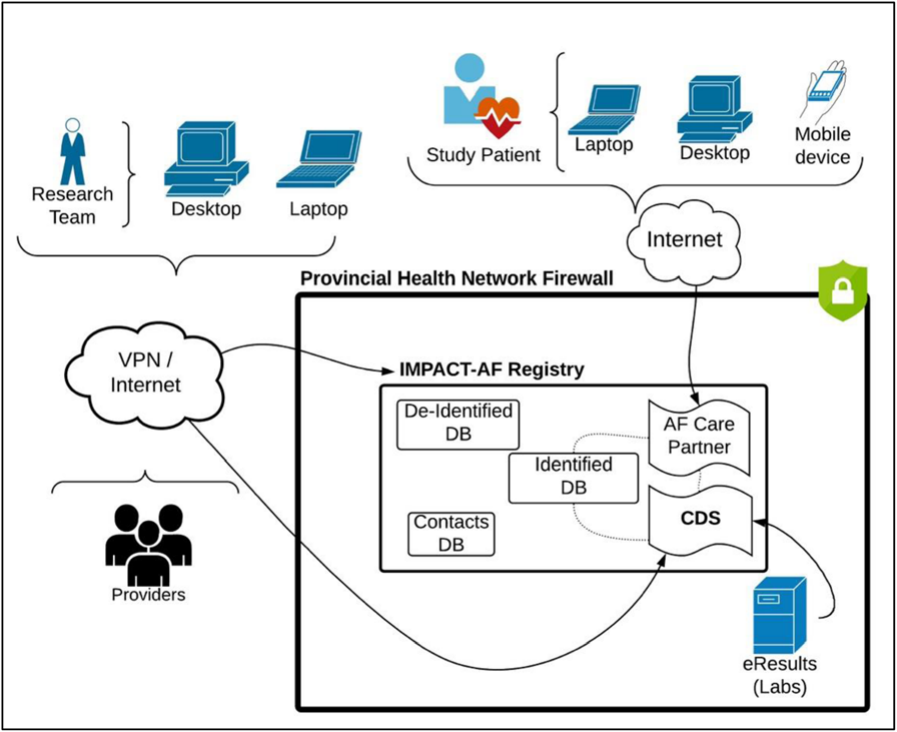
CDS information flow. DB, database; CDS, computer decision support; VPN, virtual private network

Bringing together clinical experts with a largely non-clinical health informatics team exposed many challenges (Table 1) and, partly as a result, CDS deployment took longer than projected. The most significant challenge concerned software development, which was quite complex given the objective of providing more than unidimensional functionality, such as simply supporting prescribing of antithrombotic therapy. A technology advisory committee was created to provide guidance and input into software design, and a small convenience sample of PCPs (*n* = 6) provided practical input based on pilot testing over 3 months. Figures 1 and 2 illustrate the CDS design and information flow. Programming was intricate, and the CDS needed to undergo considerable modification in response to user feedback, including updates to screen layouts and the addition of new functionalities. Despite such efforts, user assessments gathered during and at the end of the study indicated that the CDS was not user-friendly and did not create sufficient efficiencies in the management of their AF patients. Reasons cited included too many “clicks” to accomplish a given task, or repeated alerts for tasks already addressed that needed to be manually cleared, contributing to “alert fatigue” [12]. Although the clinical research team anticipated the ability to modify the CDS features and functions over time, the health informatics team favored a non-iterative approach, limiting system modification of the product once it was launched. This hampered the ability to have the software enhanced, updated, or modified to best meet end-user needs as identified overtime with an increasing number of users and ongoing CDS use.

**Table 1:** CDS development and implementation challenges and strategies

Challenges	Strategies to address challenges
Communication, including terminology (clinical- versus informatics-based)	In person meetings with written decisions to create common understanding
Timeline to develop CDS	
• Purchase and placement of hardware, privacy and ethical requirements • Availability of researchers • Simultaneous development of study database • Privacy Impact Assessments	• Stakeholder engagement, proactive communication and collaboration • Dedicated meeting times • Minimize changes to dataset, update outstanding items during data cleaning • Repeated stakeholder meetings, sought guidance from provincial experts
CDS integration	
• Proprietary Electronic Medical Record (EMR) software prohibited integration • Lack of access to “live feeds” of provincial data • Lack of integration with provincial datasets for health outcomes data	• Study abstractors pre-populated the CDS with relevant historical data for consented patients • Mimicked common EMR features in CDS (e.g., method to record medications) • Utilized same login credentials for EMR and CDS where feasible • Guided providers with minimum clinically relevant data to record in CDS • Created workaround to copy study on labs (through use of secondary ‘mailbox’) • Data gathering virtually where feasible, with in-person site visits when required
Slow Internet and CDS operating speeds	• Reduce backend platform features to improve reactivity • Focused communications on key tasks (minimum user expectation)
Supporting intervention participants	• Pre-populated CDS with relevant historical data for consented patients • Study office developed facile support tools (e.g., quick start guide), offered telephone support during office hours (8 am–4 pm) • Hired “EMR Peer Champion” to lead training/illustrate how PCPs could integrate CDS into office work flow • Focused communications on key tasks (minimum user expectation) • Completed quarterly provider check-ins • Created “How To” YouTube videos • Communicated with patients on how to use Patient Care Partner tools

During the time of CDS development, there were three popular EMRs in Nova Scotia primary care. While the CDS software was built using standardized computer programming language, it could neither be integrated within desired provincial e-health systems nor, perhaps more importantly, embedded within any of the EMRs especially due to proprietary issues, but also cost and time constraints. The lack of EMR integration meant that a greater burden of responsibility was placed on PCPs to enter relevant AF patient data such as changing medications or bloodwork results themselves in order to ensure the intended operational functionality of the CDS. The time and effort required to do this ran contrary to the intended purpose of the tool.

As noted earlier, a key CDS feature was prioritized automated alerts signaling material changes in patient clinical or biochemical profiles requiring expedited treatment modifications. Since EMR integration was not feasible, for bloodwork essential in the monitoring AF patient care, a work-around to capture test results from provincial laboratories and have them available in the CDS was created. For context, bloodwork results from provincial laboratories, the most commonly used blood collection services, are routinely available electronically to ordering physicians in Nova Scotia. Test results from the limited number of private laboratories however are not integrated into the Provincial electronic system. PCPs in the intervention arm were instructed to “copy” the study (using a dedicated identification code) on all test requisitions submitted to provincial laboratories with data relevant to study patients. The applicable CDS fields could then be auto-populated with the electronic results once posted. For those providers using private laboratories, manual entry of test results was required, creating yet another burden for CDS use. Remembering to indicate on test requisitions for consented patients that results be copied to the study was problematic, with only 29.2% (*n* = 26) doing so. An additional 10 PCPs used the manual data entry option. Thus, in total, 36 (40.4%) of 89 PCPs eligible for the intervention had recorded labs in the CDS.

The lack of EMR integration also meant that providers had to log into two separate platforms, their EMR and the CDS, to record relevant details for consented study patients. The study office guided providers on the minimum dataset required (e.g., patient blood pressure, medication or weight changes) to maximize the benefit of the CDS (run the decision rules) while minimizing provider workload. For many, double login and data entry were notably cited as impediments to optimal CDS use and as reasons for CDS provider withdrawal. Internet access and slow network broadband speeds also proved to be key challenges for successful CDS uptake, particularly in rural areas. Such areas contain just under half of the province’s population and the study purposefully sought to sample and recruit so as to reflect this.

The challenges with data integration across e-health platforms also prevented easy and timely data access, analysis and reporting. As noted, initial design plans (Fig. 1) were for the CDS to receive real-time health data (e.g., labs, echocardiogram reports, cardiovascular [CV] hospitalization and or AF-related emergency department [ED] visit data) from various sources. After ethical and privacy impact assessment, and in discussion with relevant stakeholders including provincial information technology specialists, this was not deemed to be achievable for this research initiative. The study then strove for quarterly data transfers, but ultimately this was also unfeasible due to the inability to readily link electronic provincial health datasets with the study database. As noted previously, this limited the operational functionality of the CDS (i.e., lack of new data to run the decision rules and trigger alerts based upon changing medical biochemical parameters of consented patients). In the end, the study team had to access and manually record baseline data directly into the study database from the provider’s EMRs. This activity (recording baseline data into the study database) did pre-populate each intervention provider’s CDS with historical patient data from which to begin their active PCP study phase.

In order to access patient charts and or data remotely, many steps had to be identified then taken to ensure both the ability to access and maintain the privacy and security of the required information (see Table 2). While the set-up procedure was a one-time occurrence, the processes and obstacles faced were challenging. However, having the ability to access data remotely offered several benefits, with reduced travel for study staff and less in-office disruptions for providers. In addition, the need to utilize and securely store vast amounts of paper-based study records (e.g., case report forms) was eliminated.

**Table 2:** Steps necessary for remote primary care data collection

• Abstractor laptop equipped with virtual private network (VPN) software • Approval by the provincial health information management network with login credentials (form submitted by clinic, approved by the provincial network and the new user created) • Confirmation on timelines necessary for access to be granted (specific study start and end dates communicated to each provider) • Access established for each abstractor (a unique user name, password and permissions) at each medical clinic by that clinic’s electronic medical record (EMR) lead • Coordination with clinics regarding convenient dates/times to access study patients’ charts	

Delays were experienced in retrieving 12-month patient health outcomes, specifically ED visits, hospitalizations, and especially mortality. Some of these key health outcome variables were only available from on-site reviews of hospital charts. This required considerable travel across the province, which has an area slightly larger than that of Vermont and New Hampshire combined, at the cost of human and financial resources as well as time expenditures that had not been anticipated. Thus, while the last patient completed their 12-month follow-up in January 2018, the analysis of study outcomes was delayed until the Fall of that year.

Collecting data from multiple sources (provincial systems, provider EMRs and patients themselves) created challenges of data integration and analysis. Patient-reported event timelines often did not overlap with those recorded in the PCP charts, limiting the ability to compare patient self-reported events with administrative health data. The primary source for health outcomes data were hospital-based records, accessed remotely where feasible and at times in person, as noted above. These data were cross-referenced with provincial health datasets, providers’ EMRs, and patient self-reported data for completeness.

#### Study recruitment and engagement

*Key lesson: Primary care participant recruitment was resource intensive. Significant effort (time and human resources) was required to visit medical clinics and build rapport with PCPs’ offices, including administrative staff who often play critical roles in supporting providers’ research activities.*

Based upon sample size calculations, it was estimated that upwards of 200 PCPs across the province would be required in order to meet patient recruitment goals. Looking back, this was an aggressive target considering that there were only approximately 1000 PCPs within the province at the time. Once ineligible providers were excluded (those without high-speed internet and or primarily involved in speciality work such as pediatrics, palliative care, addiction services or ED coverage), the recruitment pool was 827 (including primary care nurse practitioners). While it took 2 years to achieve (from start to final enrollment, 2014–2016), the IMPACT-AF study was successful in recruiting and randomizing 203 PCPs, representing approximately one quarter of all eligible providers in the province [6]. The recruitment strategies employed, along with their respective impact levels, are found in Table 3. The most successful strategy was resource-intensive, involving in-office small group lunch-and-learns, organized and implemented across the province. Up to 10 points of contact (including email, faxes, telephone calls, webinars, in-office visits and continuing medical education events) were required to recruit an individual provider. The most common reason for non-participation of potentially eligible providers was time constraints (due to a lack of staff, staff turnover, current participation in other research, or the provider practicing in multiple locations). Other reasons included practice size deemed to be too small, a simple lack of interest in the research topic, concerns with “double-data entry” (given that the intervention tool was not contained within the providers’ EMR) and or privacy concerns (since the study abstractors had to access and review the PCP’s charts of consented patients for relevant study data). Reasons cited for provider withdrawal over the duration of the study included lack of time or human resources to conduct planned study activities such as dual EMR/CDS login and data entry, too much effort to recruit patients and retirement or departure from the practice [6, 13].

**Table 3:** Provider identification, recruitment, and engagement strategies

**Strategies with positive impact**	
Identified a well-respected provider champion to sit on the project’s Executive Committee in order to provide real-world suggestions and guidance	
Retrieved an electronic copy of the provincial provider list from the College of Physicians and Surgeons (mailing address, telephone and fax numbers)	
Conducted a needs assessment to better understand provider challenges with AF management and use of/interest in CDS tools	
Applied for and secured approval of continuing professional development credits for participating providers	
Conducted small group virtual (webinar, teleconference) education sessions	
Conducted site visits to providers’ offices, dropping information in-person to front office staff in order to create a “friendly face” and get to know the names of clinic staff for future communications	
Conducted clinic-level lunch and learns to explain the study concept and design; over time, messaging was tailored to be more pragmatic about the benefits of participation for the provider	
Utilized peer colleague referrals (e.g., first provider within a clinic to invite their colleagues to participate)	
Offered some limited remuneration for non-accredited study activities in recognition of providers’ time	
Created a study website, including the ability for providers to register for a webinar event and view news/media articles about the study and investigator team/study office staff	
Encouraged interested community members (potential study patients) to discuss participation with their provider	
Contacted providers of those patients who called the study office wanting to participate [providers not enrolled at the time]	
**Strategies with little to no impact**	
Set-up information booths at academic conferences with promotional materials (brochure/frequently asked questions)	
Faxed all providers study information (initial invitation to all Nova Scotia providers and ongoing fax communications for recruited providers)	
Conducted local, community-based accredited education sessions to explain the study concept and design, coordinating events where possible with community-based continuing medical education leads	
Mailed information brochures to all providers	
Sent personalized communications (telephone, fax) from the lead researchers to PCPs they knew, inviting them to participate in the study	
Published communications in provincial physicians’ associations magazines and eNewsletters	
Utilized social media channels (Twitter, Facebook) to increase awareness	
Sent a communication to community-based specialist leaders to ensure they were informed about the study	
**Strategies with negative impact**	
Frequent follow-up telephone calls with PCP offices	

#### Patient recruitment and engagement

In order to meet sample size calculations, the proposed patient recruitment target was 1075. With a provincial population of just under one million, and an estimated AF prevalence of 1%, the study required approximately 10% of all patients living with AF to participate [14, 15]. For ethical reasons, PCPs were required to be the initial point of contact for patient identification. Providers were shown methods they could utilize to identify their own eligible AF patients (e.g., review of patient records or billings). The specific strategies used for patient recruitment and their impact are listed in Table 4. Traditional resources for advertising the study to patients, such as posters, pamphlets, and standardized invitation letters, were produced and shared. Early on, the research team deemed that this more passive approach would not be sufficient given the project timeline and milestone deliverables. The team also realized the critical role that front-office administrative staff can play in supporting research activities, including patient recruitment. Accordingly, primary care clinic staff were provided with training and supports in order to identify potentially eligible patients. Unplanned study resources, such as the hiring of additional study staff to provide assistance and consent patients were required to accelerate these efforts.

**Table 4:** Patient identification, recruitment, and engagement strategies

**Strategies with positive impact**	
Offered PCPs detailed instructions on methods to identify potentially eligible participants via query of the provider’s EMR using billing codes or medications fields	
Created a standardize form that providers could fax to the provincial administrative billing system which would query their historical billing codes and generate a list of potential patients to invite, with costs of this query covered by the study office	
Created and shared a telephone script which provider’s office staff could use to contact patients and obtain a verbal consent to be contacted by the study office, who could then explain the study and consent the participant	
Offered a “no-survey option” for patients who initially declined participation (i.e., highlight that the completion of patient questionnaires and the 12-month diary were voluntary)	
Employed various strategies to recognize and incentivize PCPs that were top patient recruiters (e.g., Thank you letters, study update communications, special invitation to participate in a “Canadian Science Forum” investigator event)	
Sought guidance from top recruiting PCPs on the methods they had utilized to successfully recruit their patients (these details informed revisions to various patient communication tools)	
Developed and published mainstream media communications to create awareness of the study, including multiple interviews with news outlets (television, radio) and opinion piece submissions to both local community-based and provincial newspapers	
Utilized study and non-study (e.g., stakeholder partners’) social media platforms such as Twitter, Facebook, a dedicated study website (www.impact-af.ca) and other sites (e.g., “Kijiji”) to promote the study	
Hired a public relations summer student to support communication efforts	
**Strategies with little to no impact**	
Invited consented study patients to speak with relatives and friends about the study	
Offered in-office visits by study staff to facilitate introductory and consent discussions with eligible patients	
Created a faxable letter to community pharmacies to potentially identify patients prescribed oral anticoagulant (warfarin or non-vitamin K antagonist) therapy	
Provision of pre-stuffed information packages with postage and instructions on ways to generate a mailing list that the provider could use to distribute information pamphlets	
Provision of information pamphlets and posters that could be placed in providers’ offices as well as community blood collection sites	
Created customized yet personalized letters using the provider’s office logo/letter head (with their consent) to accompany the patient information pamphlets handed and or mailed out	
Provided routine status updates to PCPs on their recruitment stats, as well as that of their peers and the study overall	
**Strategies with negative impact**	
Conducted follow-up telephone calls with study participants	

In our study, the average cluster size for active providers at 12 months (*n* = 77 usual care, *n* = 89 CDS, as per the Consort flow diagram [6]) was 6.8 patients. There were 46 PCPs (27.7%) who recruited between six and seven patient participants. Another 49 (29.5%) PCPs recruited more than six patients, while 71 (42.8%) enrolled fewer than this. Ultimately, the 1133 study patients included in the outcomes analysis represented an estimated 12% of all Nova Scotia residents with AF [6]. PCPs with past research experience or who led patient consent were top recruiters.

In this study, there was no single patient recruitment strategy that was clearly superior at maximizing patient recruitment, but rather a combination of approaches whose assortment often differed between practices. In regard to retention, there was a single issue viewed negatively by both patients and providers that led to study withdrawal. This related to what was felt to be too-frequent contact by the study office, whether by phone or email, in order to obtain follow-up information or solicit study feedback. Once this became clear, the study office attempted to limit the volume of communications as much as possible and to focus on collecting only the information deemed most essential. The aspect of the study affected was the qualitative one, with less information that aimed to assess the personal burden and economic costs of AF being passed on by patients over time.

## Discussion

Despite the many challenges faced by the IMPACT-AF research team, there were some successes, such as recruitment of highly representative samples of primary care providers and their patients with AF from within the small province of Nova Scotia, Canada, along with the rigorous assessment of a CDS tool. Disappointingly, however, clinically significant improvements in the primary study outcome (a composite of unplanned CV hospitalizations and AF-related ED visits) in favor of the CDS were not observed at 12-month follow-up [6].

Based upon our learnings, we would propose several suggestions for any investigators planning to undertake a pragmatic clinical trial of clinical decision support tools. As a fundamental first step, appropriate expertise and sufficient resources should be allocated for CDS software development, adequate feasibility testing, software updates as required, and technology support. A dedicated and robust testing and feasibility assessment phase, with an iterative design process whereby end-user feedback can be readily incorporated to improve the usefulness of the application would be highly recommended. For point-of-care CDS tools to be useful, they must meet the needs of the end users, be they physicians and or patients. Ideally, individuals with a background in both Medicine and Health Informatics should be engaged at an early stage in CDS development as they will have especially important insights to provide in regard to clinical content and software algorithms. However sophisticated its software, the success of any CDS will ultimately depend on its value to its intended users. Accordingly, effort should be invested into ensuring that it is user friendly, provides work efficiency, and has a clear and attractive interface that is easy to navigate. To this end, a representative sample of the targeted end-users (e.g., practicing primary care providers and their patients) needs to be consulted throughout the design process, allowed sufficient time to test it, and have their feedback carefully considered. Software updates should be provided as soon as needed to address any “bugs,” glitches, or other unforeseen problems negatively impacting the application. Information technology support, preferably by phone, but also via on-line means, with an easy-to-understand user manual, or a combination of some or all of the above, should be made available. Such comprehensive and timely software updates and technology support could not be provided in the IMPACT-AF study.

We also recommend that any CDS be fully integrated with, or capable of seamlessly interacting with, any related applications used by physicians or patients, such as EMRs or patient health tracking devices, to increase the likelihood of use. Some studies have shown that even when CDS software or automated alerts are fully embedded within EMRs, these features can be under-utilized [15–17]. Based upon our experience, any platform that requires double login and data entry is simply not worth pursuing as it is unlikely to be much used, if at all. This point cannot be over stressed. Despite PCPs interest, desire and willingness to participate in the research, duplicating work in the real-world setting was not sustainable for most busy clinicians. Another requirement for CDS software to function optimally is to have them integrated with as many clinical datasets as possible across a given healthcare system. Comprehensive patient care requires consideration of the individual as a whole, not just one medical condition (e.g., AF) or a component of that one condition (prescription of antithrombotic medication). In the future, CDS tools capable of integrating multiple disease guidelines as required to manage multi-morbid patients, and that can seamlessly integrate with patient health monitoring devices and eHealth platforms including EMRs, are those most worth pursuing.

The ability to have timely access, analysis and reporting of data for quality assurance and improvement initiatives, as well as for clinical research, is imperative. A field documenting patient consent for contact regarding clinical trial involvement or to allow deidentified use of their data could readily be included in the CDS. With heightened concerns over potential privacy breaches however, considerable security and precautions are required to protect patient confidentiality.

Emphasizing the potential benefits accruable to PCPs for their participation in a study of a CDS tool, rather than focusing simply on the potential to improve patient outcomes, seemed to enhance provider recruitment. In particular, highlighting the possibility that use of such decision support can result in a more efficient workflow might be an effective inducement for study enrolment. We would recommend engaging well-respected peer champions to help promote the benefits of CDS applications in enabling patient care and workflow and as well in helping with physician recruitment. Ultimately, the study office had to enhance significantly its efforts to better support providers to achieve patient recruitment targets, thus allocating adequate resources—time and human—during the study planning phase would be prudent. Given the critical role clinic administrative staff play in supporting PCPs with patient identification and recruitment, engaging and supporting these individuals is also strongly recommended.

### Limitations

The observations presented here are based upon our experiences and challenges faced within the IMPACT-AF study over time (2013–2020) conducted in Nova Scotia, Canada. Other CDS development teams may not have similar experiences, instead utilizing an iterative design process with a robust testing phase and incorporating sufficient modifications based upon end-user feedback. Certainly, the lack of facile data integration across and within health systems is common. The impacts of the recruitment strategies may vary in other jurisdictions and or be dependent upon the resources available to support them, timing of their deployment, and ongoing implementation during a clinical trial.

## Conclusion

A rapid growth in healthcare data is leading to widespread development of CDS software to analyze it, better support evidence-informed patient management and thereby improve health outcomes. Our experience of developing and implementing a pragmatic, cluster randomized controlled trial of a CDS in the real-world setting found a variety of practical issues to address if such applications are to succeed. There is a need for the allocation of adequate resources for CDS software development and updates, robust feasibility testing, as well as for primary care study recruitment. Most critical is a need for the integration of applications across e-health platforms. We hope that by sharing our experiences others can learn and achieve greater success in their future pragmatic clinical trials of CDS tools.

## Data Availability

Not applicable.

## Abbreviations

AF: Atrial fibrillation
CDS: Clinical decision support
CV: Cardiovascular
ED: Emergency department
EMRs: Electronic medical records
PCPs: Primary care providers
VPN: Virtual private network
