# Correction: Lessons learned from Integrated Management Program Advancing Community Treatment of Atrial Fibrillation (IMPACT-AF): a pragmatic clinical trial of computerized decision support in primary care

**DOI:** 10.1186/s13063-022-06484-6

**Published:** 2022-07-05

**Authors:** Joanna M. Nemis-White, Laura M. Hamilton, Sarah Shaw, James H. MacKillop, Ratika Parkash, Shurjeel H. Choudhri, Antonio Ciaccia, Feng Xie, Lehana Thabane, Jafna L. Cox, for the IMPACT-AF Investigators

**Affiliations:** 1 Principal, Strive Health Management Consulting Ltd., Halifax, Nova Scotia Canada; 2 Research Manager, QEII Health Sciences Centre, Nova Scotia Health Authority, Halifax, Nova Scotia Canada; 3 Healthy Communities Program Officer, Public Health, Nova Scotia Health Authority, Halifax, Nova Scotia Canada; 4 Family Physician, Sydney Primary Care Medical Clinic, Sydney, Nova Scotia Canada; 5 Division of Cardiology, Department of Medicine, Dalhousie University, Halifax, Nova Scotia Canada; 6 Senior Vice President and Head, Medical & Scientific Affairs, Bayer Inc, Mississauga, Ontario Canada; 7 Director & Head, Medical Affairs – Cardiovascular Medicine, Bayer Inc, Mississauga, Ontario Canada; 8 Professor, Department of Health Research Methods, Evidence, and Impact, McMaster University, Hamilton, Ontario Canada; 9 Centre for Health Economics and Policy Analysis, McMaster University, Hamilton, Ontario Canada; 10 Vice President, Research, St. Joseph’s Healthcare, Hamilton, Ontario Canada; 11 Professor, Departments of Anesthesia/Pediatrics, McMaster University, Hamilton, Ontario Canada; 12 Director, Biostatistics Unit, Centre for Evaluation of Medicine, McMaster University, Hamilton, Ontario Canada; 13 Senior Scientist, Population Health Research Institute (PHRI), Hamilton Health Sciences, McMaster University, Hamilton, Ontario Canada; 14 Department of Community Health and Epidemiology, Dalhousie University, Halifax, Nova Scotia Canada; 15 Heart and Stroke Foundation of Nova Scotia Endowed Chair in Cardiovascular Outcomes Research, Halifax, Nova Scotia Canada

**Correction: Trials 22, 531 (2021)**

10.1186/s13063-021-05488-y

Following the publication of the original article [1], we were notified that Fig. 1 needs to be replaced.

Incorrect Fig. 1:

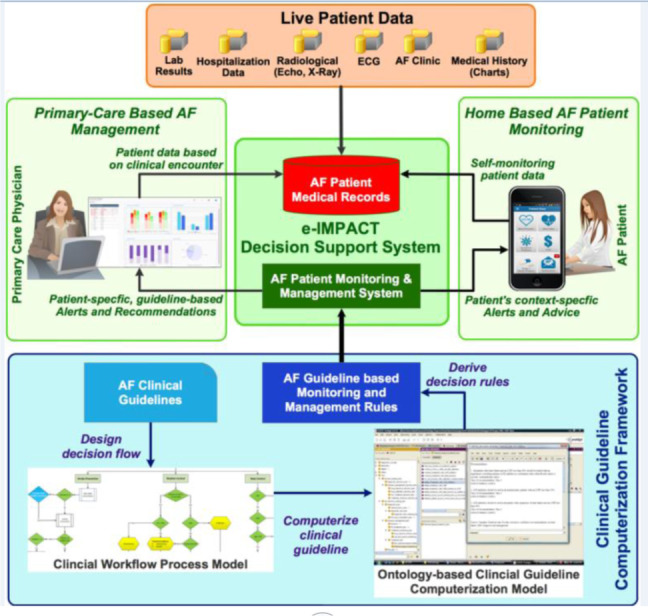



Corrected Fig. 1:

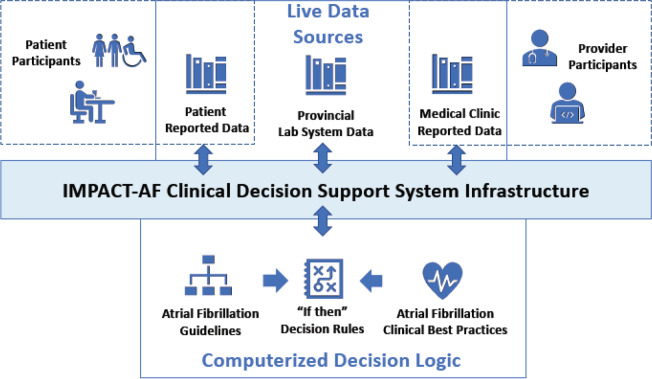



The original article has been corrected.
